# Molecular and biochemical components associated with chilling tolerance in tomato: comparison of different developmental stages

**DOI:** 10.1186/s43897-024-00108-0

**Published:** 2024-09-05

**Authors:** Maria Dolores Camalle, Elena Levin, Sivan David, Adi Faigenboim, Majid R. Foolad, Amnon Lers

**Affiliations:** 1 Department of Postharvest Science, Volcani Institute, Agricultural Research Organization, Rishon LeZion, Israel; 2 Robert H. Smith Faculty of Agriculture Food and Environment, The Robert H. Smith Institute of Plant Sciences and Genetics in Agriculture, The Hebrew University of Jerusalem, Rehovot, Israel; 3 Institute of Plant Sciences, Volcani Institute, Agricultural Research Organization, Rishon LeZion, Israel; 4 Department of Plant Science, The Pennsylvania State University, University Park, PA USA

**Keywords:** Postharvest, Tomato, Storage, Cold, Chilling

## Abstract

The cultivated tomato, *Solanum lycopersicum*, is highly sensitive to cold stress (CS), resulting in significant losses during cultivation and postharvest fruit storage. Previously, we demonstrated the presence of substantial genetic variation in fruit chilling tolerance in a tomato recombinant inbred line (RIL) population derived from a cross between a chilling-sensitive tomato line and a chilling-tolerant accession of the wild species *S. pimpinellifolium.* Here, we investigated molecular and biochemical components associated with chilling tolerance in fruit and leaves, using contrasting groups of “chilling tolerant” and “chilling sensitive” RI lines. Transcriptomic analyses were conducted on fruit exposed to CS, and gene expressions and biochemical components were measured in fruit and leaves. The analyses revealed core responding genes specific to either the cold-tolerant or cold-sensitive RI lines, which were differentially regulated in similar fashion in both leaves and fruit within each group. These genes may be used as markers to determine tomato germplasm cold tolerance or sensitivity. This study demonstrated that tomato response to CS in different developmental stages, including seedling and postharvest fruit, might be mediated by common biological/genetic factors. Therefore, genetic selection for cold tolerance during early stages of plant development may lead to lines with greater postharvest fruit chilling tolerance.

**Supplementary Information:**

The online version contains supplementary material available at 10.1186/s43897-024-00108-0.

## Core

We have employed an interspecific RIL population of tomato and demonstrated the presence of substantial genetic variation in fruit chilling tolerance in the population. Molecular and biochemical studies identified core responding genes specific to either the cold-tolerant or cold-sensitive RI lines, which were differentially regulated in a similar fashion in both fruit and leaves within each group. This study revealed that tomato response to cold stress in different developmental stages, including seedling and postharvest fruit, might be mediated by common biological/genetic factors.

## Gene & accession numbers

Gene & accession numbers information on gene & accession numbers can be found in Supplementary Tables 4 & 6. All the gene information is provided in the Sol Genomics network database (https://solegenomics.net).

## Introduction

Low temperatures negatively affect cold-sensitive crops by slowing growth and development, resulting in significant reduction in yield (Ding et al. 2019; Zhang et al. 2019). Specifically, cold stress (CS) may cause reduced plant growth and flower production, flower abortion, reduced fruit set, and injuries to the developing fruit, resulting in fewer harvestable and marketable fruit (Hedhly 2011; Thakur et al. 2010; Zinn et al. 2010). Further, additional fruit may be lost post harvest due to unsuitable storage conditions. The ability to store postharvest fruit at low temperatures, however, may conserve fruit quality by lowering respiration rate and other basic metabolic processes involved in senescence and ripening, leading to less deterioration. Many vegetable and fruit crops, including tomatoes, peppers, and avocados, are cold-sensitive during postharvest storage and suffer from temperatures below their chilling tolerance threshold and may exhibit chilling injuries, such as skin pitting, internal or surface browning, water-soaked tissue, abscission, and decay development (Kratsch and Wise 2000; Sevillano et al. 2009; Valenzuela et al. 2017); such injuries may also lead to susceptibility to diseases during storage.

The primary physiological causes of chilling injuries include modifications or damages to cell walls and membranes, which would disrupt their integrity or functionality, leading to ion leakage across membranes (Orvar et al. 2000; Ruelland and Zachowski 2010). Membrane damage may set off cascades of secondary responses, including ethylene production, increased respiration, reduced photosynthesis, interference with energy production, and accumulation of toxic compounds (Kratsch and Wise 2000). Consequently, the accumulation of reactive oxygen species (ROS) (Ruelland et al. 2009; Suzuki and Mittler 2006) and lipid peroxidation (Liu et al. 2020) would alter cellular homeostasis, leading to plant cell death.

The ability of certain plant species or genotypes within species to endure CS lies, at the molecular level, in the early activation of specific signals such as the burst of calcium and production of ROS, which would trigger the MAPK (mitogen-activated protein kinase) signal transduction cascade (Yang et al. 2010; Yuan et al. 2018; Zhu 2016). This cascade eventually initiates cold-responsive transcriptional signal transduction, activating stress adaptation and tolerance-response genes. At the biochemical level, upon exposure to CS, starch degradation leads to accumulation of soluble sugar such as maltose and glucose (Tarkowski and Van den Ende 2015, Zhao et al. 2019); this accumulation, in turn, restores osmotic balance. In addition, under CS, anthocyanin production enhances plant antioxidant capacity, leading to protection against oxidative damages induced by ROS production (Naing and Kim 2021; Xu et al. 2023). It is essential, therefore, to recognize that factors contributing to CS responses are multifaceted, which could encompass single metabolites and individual signaling pathways or complex interactions, and can differ among plant species, genotypes, and in different developmental stages.

To investigate the complexity of adaptive mechanisms of CS response in tomato, earlier (David et al. 2022) we employed a recombinant inbred line (RIL) population previously developed from a cross between *Solanum pimpinellifolium* L. accession LA2093 and tomato breeding line NC EBR1 (Ashrafi et al. 2009) and subsequently genetically mapped with more than 144,000 SNP markers (Gonda et al. 2019). Accessions of the wild S. *pimpinellifolium* species can be found across a vast geographic area from Ecuador to southern Peru (Gibson and Moyle 2020; Warnock 1991). This region has diverse environmental conditions, ranging from high-altitude chilly area in the Andes, to low-altitude coastal deserts and rainforests near the Pacific Ocean. Therefore, accessions within S. *pimpinellifolium*, are valuable sources of beneficial genes and traits, including resistance to diseases (e.g. early blight, late blight and bacterial canker), tolerance to abiotic stresses (e.g. drought salt and cold), and high fruit quality (Ashrafi et al. 2012; Foolad et al. 1998, 2001; Kinkade and Foolad 2013; Rao et al. 2012; Wang et al. 2020). In the present study, we have utilized selected lines within the RIL population, which were previously identified and characterized as “cold-tolerant” or “cold-sensitive” group based on their postharvest fruit response to cold storage (David, et al. 2022). We have employed transcriptomic analyses to investigate the complexity of adaptive mechanisms associated with the response to low temperatures by comparing differential gene expression between the two RIL groups. We have identified candidate genes with possibly key roles in conferring cold tolerance. Additionally, we have compared the cold response of fruit with that of vegetative tissue taken from young tomato plants. The overall results support the involvement of common genetic factors contributing to cold/chilling tolerance in the leaf tissue and postharvest fruit.

## Results

### Differential expression of genes in fruit of the cold-tolerant and cold-sensitive RILs

For the transcriptomic analysis of fruit response to CS, we included 3 cold-tolerant (#47, 65, 99) and three cold-sensitive (#71, 135, 150) RILs as determined in our previous study (David, et al. 2022). For this analysis, we strictly required that the measured differential expression (DE) was observed in all 3 cold-tolerant *versus* all 3 cold-sensitive RILs; this allowed us to identify genes whose expressions significantly differed between the two classes after applying the CS (Fig. S1). Principal component analysis (PCA) revealed significant differences in gene expressions (GE) between 24 h CS treatment and that of either 2 h CS, or control treatment in all 6 RILs, indicating that most DE occurred after 24 of CS exposure (Fig. S2). Further, the PCA revealed significant differences in GE between the two classes of RILs after 24-h exposure to CS (Fig. S2).

**Fig. 1:**
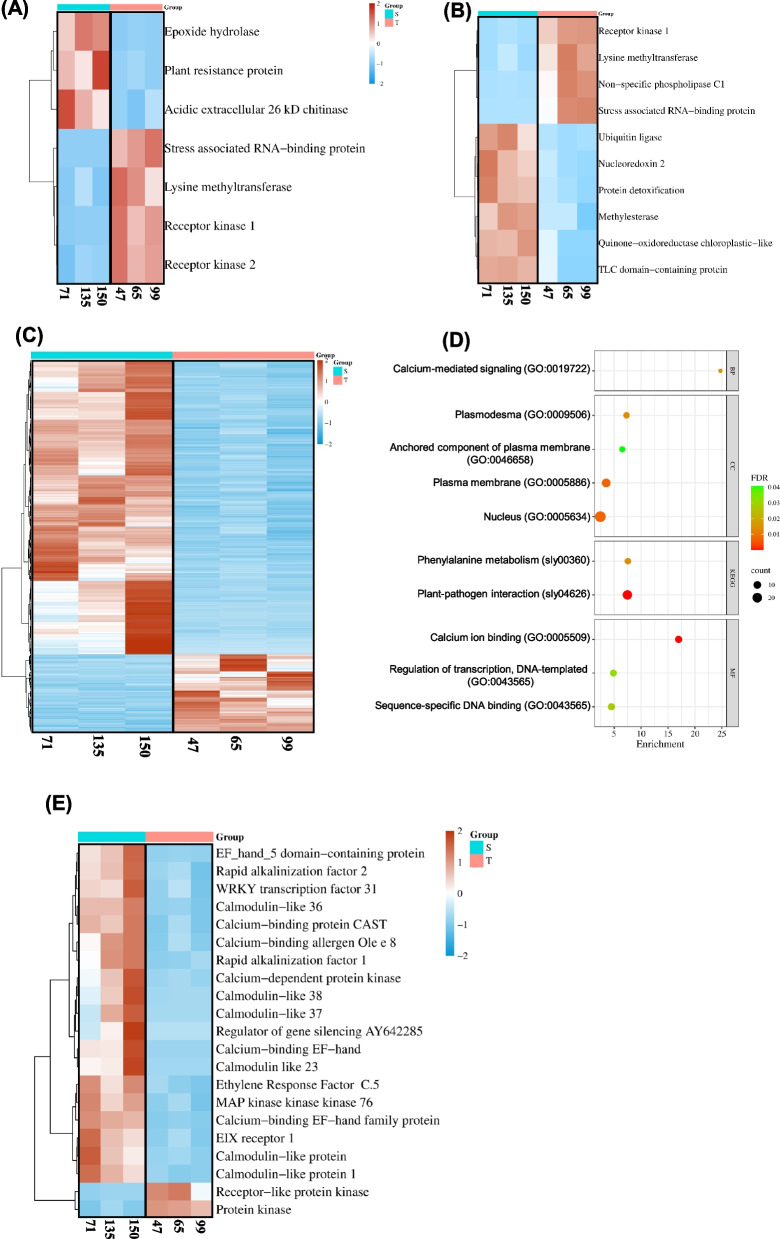
Overview of the transcriptomics of fruit response to cold stress in sensitive (5, 135, 150) and tolerant (47,65, 99) RILs. **A** Heat map showing the expression of differentially regulated genes (DEGs) among sensitive and tolerant RILs before cold stress. **B** Heat map showing the expression DEGs among sensitive and tolerant RILs after two hours of exposure. **C** Heat map showing the expression of 486 DEGs among sensitive and tolerant RILs after twenty-four-hour exposure to cold stress. **D** Gene ontology (GO) enrichment analysis of 486 DEGs from tomato fruit after twenty-four-hour exposure to cold stress using KOBAS. **E** The highly expressed 21 genes linked with calcium-mediated signaling among the 486 DEGs are presented. In the heat map, the dark red color denotes the highly up-regulated expression, and the sky-blue color denotes the down-regulated expression. S, cold-sensitive RILs, T, cold tolerant RILs. DEGs analysis was performed with DESeq2 R package (twofold, FDR < 0.05)

Before application of CS, there were significant (twofold, FDR < 0.05) differences between the two selected classes in the expression of 7 genes, including 4 genes whose expressions were higher in the cold-tolerant class (2 of which showing homology to receptor kinases involved in sensing and signal transduction of abiotic stresses (see discussion), and 3 genes whose expressions were higher in the cold-sensitive RILs (showing homologies to proteins with stress defense functions) (Fig. 1A and Supplementary Table S2). After 2-h exposure to CS, 10 genes were differentially expressed in the cold-tolerant and cold-sensitive RILs, of which 4 exhibited significantly higher GE in the cold-tolerant RILs and 6 exhibited significantly higher GE in the cold-sensitive RILs (Fig. 1B and Supplementary Table S3); the possible functions of these genes and their encoded proteins are discussed below. After 24 h exposure to CS treatment, a total of 486 genes exhibited significantly (twofold, FDR < 0.05) different expressions in the cold-tolerant and cold-sensitive RILs (Supplementary Table S4), including 385 genes with significantly higher and 101 genes with significantly lower GE in the cold-sensitive than in the cold-tolerant RILs (Fig. 1C and Supplementary Table S4). Enrichment analyses, employing the Gene Ontology (GO) and Kyoto Encyclopedia of Genes and Genomes (KEGG) pathways, determined that the group of 24 h DEGs (486 genes) was enriched for eight GO terms, including Biological Process (BP) calcium-mediated signaling, and Molecular Function (MF) calcium ion binding. The two KEGG enriched pathways were phenylalanine metabolism and plant-pathogen interactions (Fig. 1D).

Most of the DEGs associated with calcium mediated signaling were upregulated in the cold-sensitive RILs, excluding Solyc05g013320 and Solyc02g072440, which show homology to protein kinase and receptor-like protein kinase, respectively and were upregulated in the cold-tolerant RILs (Fig. 1E and Supplementary Table S5).

### Fruit transcriptomic response to chilling stress during postharvest storage

**Fig. 2:**
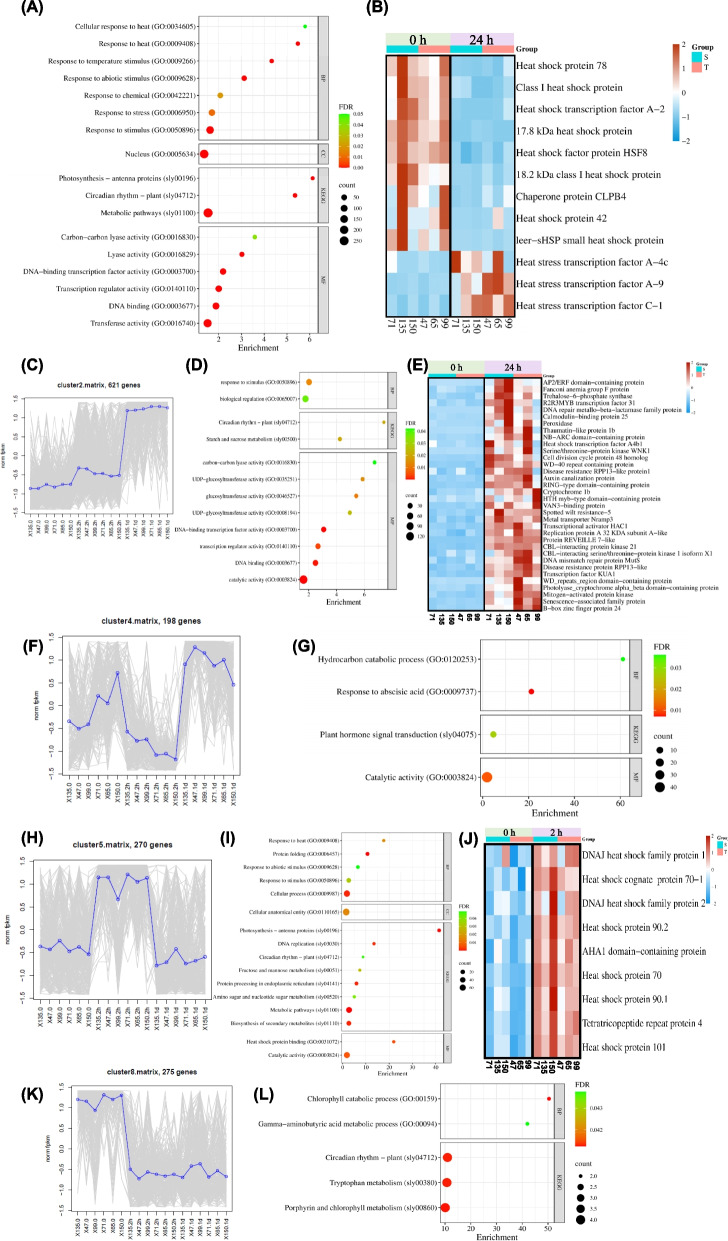
Common transcriptomic response of all six RIL fruits following 24 h exposure to 1.5°C temperature. All 2454 cold-responsive DEGs identified in all six RIL fruits were bioinformatically analyzed. **A** Bubble plot displaying GO classification and KEGG categories classifications of commonly expressed functionally annotated 2454 DEGs from all six RILs cold-sensitive (5, 135 and 150) and cold tolerant (47,65 and 99). **B** Heatmap analysis presenting common response of genes included in BP term—response to heat. **C** Expression pattern of cluster 2, 621 DEGs. **D** Bubble plot displaying GO classification and KEGG categories classifications for functionally annotated DEGs commonly expressed and grouped in cluster 2. **E** Heatmap analysis presenting common response of genes included in the GO term—response to stimuli included in cluster 2. **F** Expression pattern of cluster 4, 198 DEGs. **G** Bubble plot displaying GO classification and KEGG categories classifications for functionally annotated DEGs commonly expressed and grouped in cluster 4. **H** Expression pattern of cluster 5, 270 DEGs. **I** Bubble plot displaying GO classification and KEGG categories classifications for functionally annotated DEGs commonly expressed and grouped in cluster 5. **J** Heatmap analysis presenting common response of genes included in the GO term—protein folding included in cluster 5. **K** Expression pattern of cluster 8, 275 DEGs. **L** Bubble plot displaying GO classification and KEGG categories classifications for functionally annotated DEGs commonly expressed and grouped in cluster 8. In heatmaps, the dark red color denotes up-regulated DEGs, and the sky-blue color denotes down-regulated DEGs. GO enrichment analysis of 2454 genes were done using KOBAS and PANTHER. DEGs analysis was performed with DESeq2 R package (twofold, FDR < 0.05)

In addition to genes that were differentially expressed in response to CS in opposite directions in the cold-tolerant (3 RILs) *vs.* cold-sensitive class (3 RILs), a total of 2454 genes were identified that were differentially expressed (twofold, FDR < 0.05) in the same way in both classes (Supplementary Table S6). We conducted GO annotation analysis to elucidate these genes' potential functions. Biological Process (BP) general enriched terms for these transcripts included response to general stimuli, including abiotic stressors such as cold and heat (Fig. 2A). Molecular Function (MF) enriched GO terms included transferase activity, DNA binding, transcription regulator activity, DNA-binding transcription factor activity, lyase activity, and carbon–carbon lyase activity (Fig. 2A). Cellular Component (CC) enriched GO terms included only nucleus (Fig. 2A). KEGG pathway analysis revealed that CS affected significantly general metabolic pathways and circadian rhythm with photosynthesis, where antenna proteins term was mostly enriched (Fig. 2A). According to the analysis, most genes associated with response to heat stress were downregulated in both classes after 24 h of CS treatment, while 3 heat stress transcription factors genes were upregulated (Fig. 2B and Supplemental Table S7). To identify genes that had same patterns of DE following exposure to CS, the 2454 DEGs were subjected to cluster analyses. Four clusters (#2, 4, 5, and 8) out of the 8 K-means clusters generated included genes with clear and similar regulation patterns in all 6 RILs following exposure to CS (Figure 2 and Supplementary Table S6). GO analysis was performed for the included genes to identify key biological processes represented with each cluster. Cluster 2, consisting of 621 genes, exhibited gradual induction of GE following exposure to CS treatment, reaching maximal levels after 24 h CS exposure (Fig. 2C). GO analysis of cluster #2 genes revealed two BP terms, response to stimulus, and biological regulation. Eight MF terms were identified, of which three could be related to regulation of gene expression, and 3 terms represented the transfer of saccharide moieties. The KEGG pathway analysis revealed enrichment of two terms, circadian rhythm, and starch and sucrose metabolic pathways (Fig. 2D). Further investigation into the genes included in BP responses to stimuli, revealed regulatory genes which could be involved in CS response (Fig. 2E and Supplemental Table S8). Cluster 4 included 198 genes whose expression exhibited transient reduction following 2-h exposure to CS and then increased upon 24-h CS exposure (Fig. 2F). Cluster 4 GO analysis identified enrichment of two BP terms, hydrocarbon catabolism, and response to abscisic acid. (Fig. 2G). KEGG pathway analysis revealed enrichment for plant hormone signal transduction (Fig. 2G). Cluster 5 included 270 genes, whose expression exhibited transient induction following 2-h exposure to the CS and then decreased to the original level after 24-h CS exposure (Fig. 2H). GO analysis of this cluster revealed 5 BP terms, among which response to heat, protein folding, and response to abiotic stimuli were mostly enriched (Fig. 2I). In the MF, 2 terms were identified of which heat shock protein binding was highly enriched (Fig. 2I). KEGG pathway analysis revealed significant enrichment for 8 pathways, with photosynthesis-antenna proteins were mainly enriched (Fig. 2I). After exposure to CS for 2-h, *Hsps* genes included in the BP term Protein Folding were generally upregulated compared to the control (Fig. 2J and Supplementary Table S9). Cluster #8 included 275 genes, whose expression exhibited reduction following 2-h exposure to CS and which remained low after 24 h CS exposure (Fig. 2K). The GO analysis of cluster 8 genes revealed 2 BP terms, among which the chlorophyll catabolic process was highly enriched (Fig. 2L). KEGG pathway analysis showed enrichment in circadian rhythm, tryptophan metabolism, and porphyrin and chlorophyll metabolism (Fig. 2L).

### Correlation between fruit and vegetative tissue responses to chilling stress

**Fig. 3:**
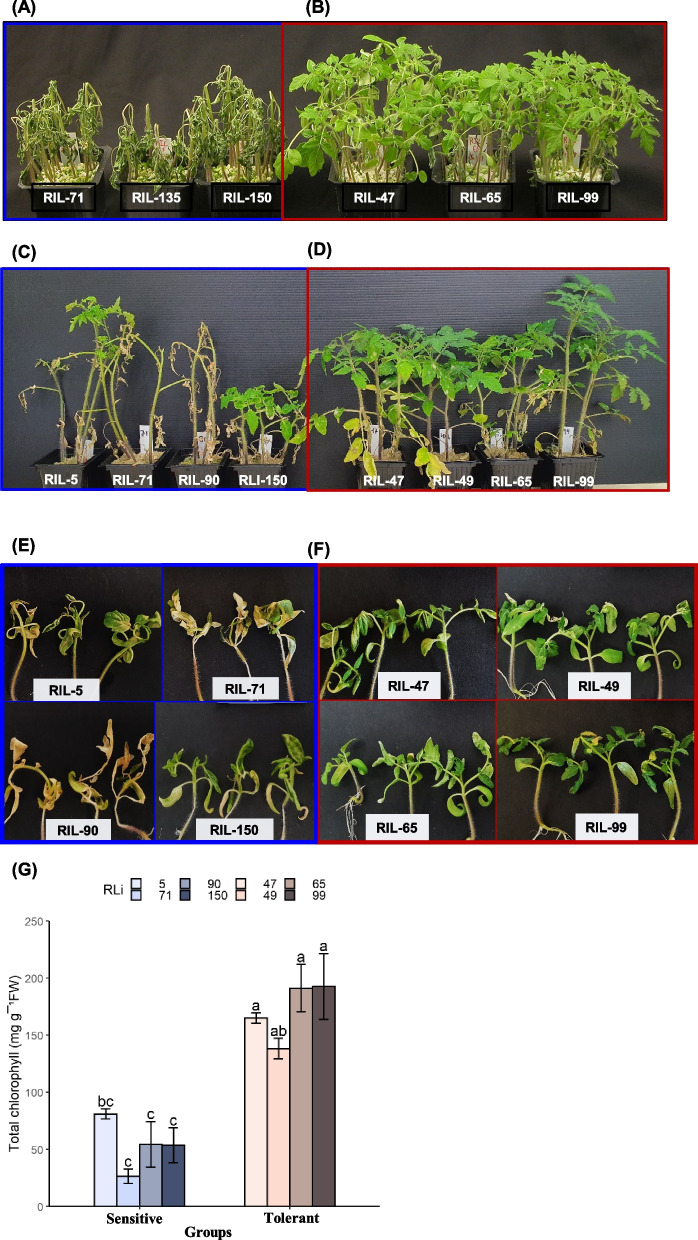
Susceptibility to cold stress of vegetative tissue in different tomato RILs. **A**, **B** Cold sensitive (71, 135, 150) and cold tolerant (47, 65, and 99) RILs were grown in perlite, and young plants (~ 30 days old; ~ 20–30 cm tall) were incubated at 1.5°C in the light for three days. Cold sensitive (5, 71, 90, 150) and cold tolerant (47, 49, 65, 99) RILs were grown in sand soil (**C**, **D**) or MS plates (~ 10 days old; ~ 8–10 cm tall) (**E**, **F**), and young plants were incubated in 1.5°C for 24 h followed with growth recovery at 25°C for 10 days. **G** Total chlorophyll content after 10 days of cold recovery of MS-grown plants. Bars with different letters indicate significant differences between sensitive and tolerant lines. One-way ANOVA *p* ≤ 0.05, as determined by Turkey-Kramer HSD

When the perlite-grown seedlings (Exp. 1) were moved from the 3-day growth at 1.5 °C to optimal temperature, the 3 cold-tolerant RILs (lines previously determined to be chilling tolerant based on their postharvest fruit) recovered quickly and continued normal growth, whereas the 3 chilling-sensitive RILs collapsed (Fig. 3A and B). Similarly, for the soil-grown plants (Exp. 2), when the seedlings were moved from 1.5 °C to optimal temperature, the 4 cold-tolerant RILs (47, 49, 65 and 99) exhibited much better survival than the 4 cold-sensitive RILs (5, 71, 90 and 150) following 10-day recovery under optimal temperature (Fig. 3C and D; Supplementary data Fig. S3A and B). In the latter experiment, all leaves of the sensitive RILs 71 and 90 turned brown, dried out and wrinkled, while for sensitive RILs 5 and 150 mainly the lower leaves dried out and wrinkled though damage was observed in all leaves (Fig. 3C and D; Supplementary data Fig. S3A). In contrast, the 4 cold-tolerant RILs (47, 49, 65 and 99) withstood the CS very nicely, and most of their leaves exhibited good recovery after moving to optimal conditions (Fig. 3D; Supplementary data Fig. S3B). Further, in the 10-d-young-seedlings grown in MS plates experiments (Exp. 3), clear differences were observed between the tolerant and sensitive RILs. Following removal of the seedlings from the CS treatment to normal temperature, the tolerant RILs remained green and viable whereas sensitive RILs exhibited yellowing with reduced survival (Fig. 3E and F). In the tolerant RILs 47, 49, 65 and 99, significantly higher chlorophyll levels were observed compared to the sensitive RILs, confirming their ability to withstand the CS better (Fig. 3G).

### Physiological and biochemical responses to cold stress in leaves of the cold-tolerant and cold-sensitive RILs

**Fig. 4:**
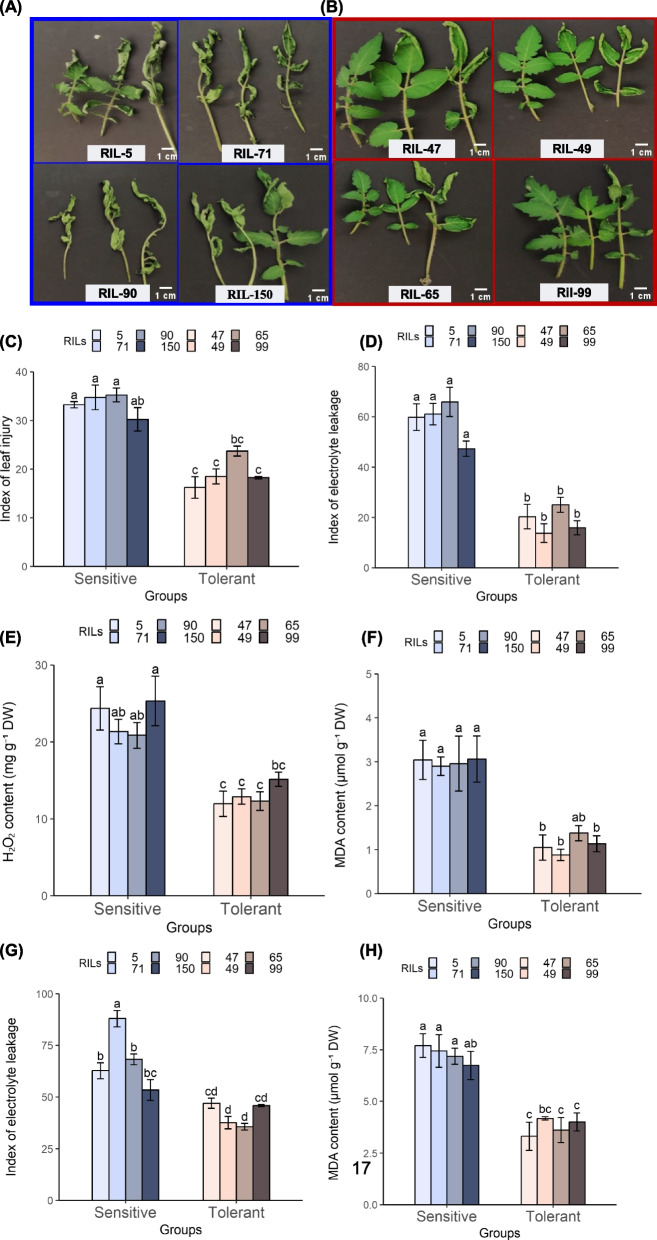
Physiological and biochemical markers indicative of chilling injury development in leaves of different tomato RILs. The different parameters were measured in soil-grown plants (~ 30 days old), including cold-sensitive (5, 71, 90, 150) or cold-tolerant (47, 49, 65, 99) RILs. **A**-**C** The impact of cold treatment on the index of leaf injury. **D** index of electrolyte leakage. **E** H_2_O_2_ content. **F** MDA content. Additionally, (**G**) the index of ion leakage and (**H**) MDA content were measured in MS-grown seedlings (~ 10 days old) following cold stress. Data are means ± SE, *n* = 4; biological replicates. Different lowercase letters indicate significant differences between sensitive and tolerant lines. One-way ANOVA *p* ≤ 0.05, as determined by Turkey-Kramer HSD

The initial visual assessment of plants following their exposure to CS indicated clear differences between the "cold-tolerant" and "cold-sensitive" RILs. Leaves of the 4 sensitive RILs exhibited higher dehydration and curling than leaves of the 4 tolerant RILs (Fig. 4A and B), and the CI index was significantly higher in the sensitive RILs (Fig. 4C). Differences between the two classes were further examined by measuring various physiological and biochemical parameters associated with chilling injury. Electrolyte leakage values were significantly higher in leaves of the cold-sensitive RILs, approximately twofold higher, than in leaves of the cold-tolerant RILs (Fig. 4D and Supplementary data Fig. S4A). The H_2_O_2_ content was significantly higher in leaves of the cold-sensitive than in leaves of the cold-tolerant RILs (Fig. 4E). Levels of malondialdehyde (MDA; lipid peroxidation product) were higher in the cold-sensitive than in the cold-tolerant RILs (Fig. 4F and Supplementary Fig. S4B). Further, the MS-grown 10-d-young seedlings (Exp. 3) also exhibited both visual (Fig. 3E and F) and physiological differences (Fig. 4G and H) when compared the cold-tolerant *vs.* cold-sensitive RILs. The electrolyte leakage index was generally higher in the cold-sensitive than in the cold-tolerant RILs, though the differences between the sensitive RIL 150 and tolerant RILs 47 and 99 were not statistically significant (Fig. 4G). MDA content was higher in the sensitive than in the tolerant RILs (Fig. 4H) for the soil-grown plants.

### Changes in starch and sugar levels in responses to chilling stress in the cold-tolerant and cold-sensitive RILs

**Fig. 5:**
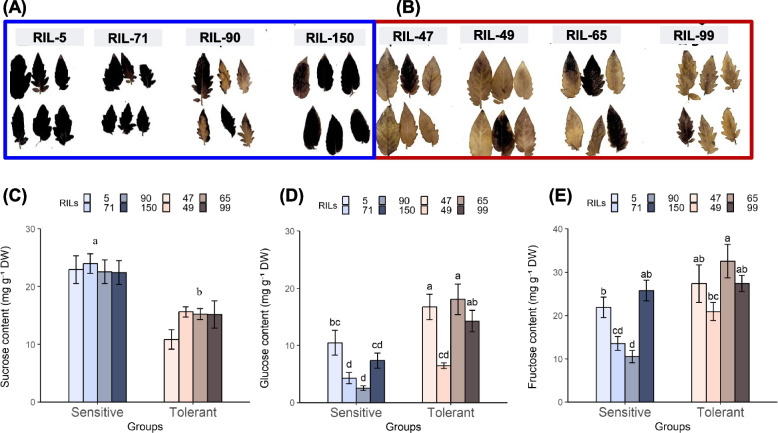
Changes in starch and sugar levels in cold-tolerant and sensitive RILs during exposure to cold stress. Sugar content was measured in leaves of soil-grown plants (~ 30 days old), including cold-sensitive (5, 71, 90, 150) or cold-tolerant (47, 49, 65, 99) RILs following 24 h in 1.5°C. **A** and **B** starch content; (**C**) sucrose content; (**D**) glucose content; and (**E**) fructose content. Data are means ± SE, *n* = 4; biological replicates. Bars with different letters indicate significant differences between sensitive and tolerant lines. One-way ANOVA *p* ≤ 0.05, as determined by Turkey-Kramer HSD and T_test (sucrose)

In the soil-grown plants (Exp. 2), after 24-h exposure to CS starch level was higher in the cold-sensitive than in the cold-tolerant RILs (Fig. 5A and B), whereas in the control plants (not exposed to cold) the starch levels were similar in both groups (Supplementary data Fig. S5A and B). Sucrose levels were significantly higher in the cold-sensitive than in the cold-tolerant RILs after 24-h exposure to CS (Fig. 5C). In contrast, glucose levels were significantly higher in 3 of the cold-tolerant RILs (47, 49 and 65) compared to all 4 cold-sensitive RILs (5, 71, 90 and 150) (Fig. 5D). Fructose levels were generally higher in the cold-tolerant RILs, though the differences were not significant between the two groups (Fig. 5E).

### Similar gene expression patterns in fruit and leaves in responses to chilling stress

**Fig. 6:**
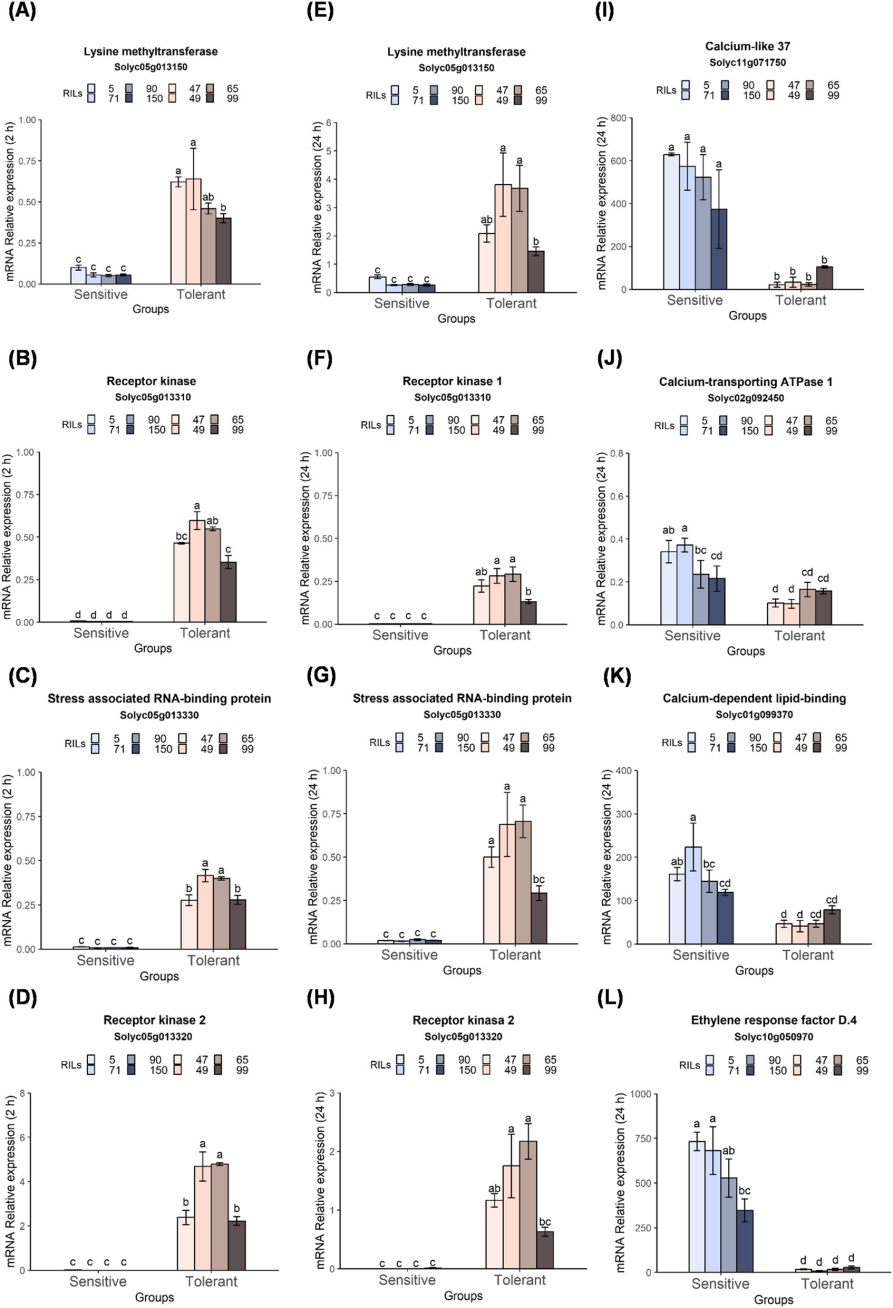
Expression of specific genes in leaves of cold-tolerant or cold-sensitive RILs following by cold stress. Gene expression was measured using RT-qPCR in leaves of cold-sensitive (5, 71, 90, 150) and cold-tolerant (47, 49, 65, 99) RILs. Soil-grown plants (~ 30 days old) were exposed to cold stress at 1.5ºC for 2 or 24 h. Data are means ± SE (*n* = 3 biological replicates). Bars with different letters indicate significant differences between sensitive and tolerant lines. One-way ANOVA *p* ≤ 0.05, as determined by Turkey-Kramer HSD

Several of the genes that were differentially expressed (DEGs) in fruit of the cold-tolerant and cold-sensitive RILs when exposed to CS (described above), exhibited similar expression patterns in leaves of the two RIL groups in response to CS. Specifically, 4 genes whose expressions were higher in fruit of the cold-tolerant than the cold-sensitive RILs, including Solyc05g013150 (with homology to lysine methyltransferase), Solyc05g013310 (with homology to receptor kinase 1), Solyc05g013330 (with homology to stress-associated RNA-binding protein), and Solyc05g013320 (with homology to receptor kinase 2), also exhibited significantly higher expressions in leaves of the cold-tolerant RILs following 2-h (Fig. 6A-D) or 24-h exposure to CS (Fig. 6E-H). Similarly, 4 genes that exhibited higher expression in fruit of the cold-sensitive than the cold-tolerant RILs, including Solyc11g071750 (with homology to calmodulin-like 37), Solyc02g092450 (with homology to calcium-transporting ATPase), Solyc01g099370 (with homology to calcium-dependent lipid binding), and Solyc10g050970 (with homology to ethylene response factor D.4), also exhibited higher expression in the leaves of the cold-sensitive RILs (Fig. 6I-L).

## Discussion

In this study, we utilized a RIL population (*n* = 148) that was previously developed from a cross between cultivated tomato breeding line NC EBR-1 and *S. pimpinellifolium* accession LA2093 (Ashrafi, et al. 2009), and subsequently genetically mapped with more than 144,000 SNP markers (Gonda, et al. 2019). The availability of marker genotypes for this RIL population (NCBI archive project number PRJNA449767) constitutes a significant advantage as described (Gonda et al. 2019). A recent screening of fruit of the 148 RILs during cold storage indicated the presence of significant phenotypic variation in postharvest fruit chilling tolerance in the RIL population (David, et al. 2022). In the screening study, 2 extremely contrasting groups (the two tail ends of the response distribution) of the RIL population were identified: 1) a “chilling-tolerant” group, including RILs 47, 49, 65 and 99, and 2) a “chilling-sensitive” group, including RILs 5, 71, 90, 135 and 150 (David, et al. 2022). In the present study, the two RIL groups were further studied to explore the molecular and biochemical aspects of the variation in the fruit and leaves of the selected RILs under chilling stress (CS). Additionally, we examined the presence of correlation between chilling tolerance during postharvest fruit storage and cold tolerance during vegetative growth stage.

The stringent screening we applied in our bioinformatic analyses of transcriptomic data, when comparing the two RIL groups, limited the number of identified differentially expressed genes (DEGs), but it raised the confidence in determining possible links between the DEGs and CS responses. Before fruit exposure to CS, only 7 genes were identified as DEGs between the two RIL groups (Fig. 1A), for most of which no information is available in the tomato database. For these genes, we utilized the Arabidopsis bioinformatic database to look for known or suggested functions of the most similar proteins. Among the 7 DEGs, 3 genes had higher transcript levels in the cold-sensitive RILs, including 1) Solyc05g054350, with a significant amino acid (AA) sequence homology to the Arabidopsis Epoxide Hydrolase (EH) enzyme, which is involved in the synthesis of poly-hydroxylated cutin monomers (Pineau et al. 2017) and autophagosome formation (Wang et al. 2019); 2) Solyc05g053980, with a limited homology to the disease resistance protein CC-NBS-LRR family, which comprises the largest class of plant disease resistance genes (Tan et al. 2007); in rice, a novel CC-NBS-LRR-like protein was found to interact with a calmodulin-like (CML) protein and together suggested to be involved in CS signaling and response (Yang et al. 2018); and 3) Solyc02g082920, with a high homology to an acidic extracellular chitinase enzyme; while chitinases are mainly known for their involvement in pathogen response, these enzymes are also involved in abiotic (including cold) stress response (Grover 2012; Kashyap and Deswal 2017). The remaining 4 genes, of the 7 pre-CS exposure DEGs, had higher expression in the cold-tolerant RILs. These included: 1) Solyc05g013330, encoding for stress-associated RNA-binding protein that shows homology to the Arabidopsis stress associated RNA-binding protein 1 (SRP1), which is a C2C2-type zinc-finger protein that binds RNA and has a role in response to ABA (Hou et al. 2017); zinc finger-containing RNA-binding proteins were previously demonstrated to be involved in plant cold tolerance (Kim et al. 2010, 2005; Kim and Kang 2006); 2) Solyc05g013150, encoding for lysine methyltransferase (LSMT), which is similar to the Arabidopsis LSMT-like protein, whose primary soluble physiological substrates are chloroplastic fructose 1,6-bisphosphate aldolases (FBA) (Mininno et al. 2012); FBA is a key enzyme in photosynthesis and was reported to be associated with CS response and tolerance in different plant species including tomato (Cai et al. 2018; Mu et al. 2021; Yu et al. 2022b); and 3 and 4) Solyc05g013310 and Solyc05g013320, identified as receptor kinases, and are tandemly arranged in the tomato genome and their encoded proteins have high homology to the Arabidopsis receptor-like protein kinases of the HERK family; HERKs are major sensors in various signal transduction pathways, including plant responses to drought, salt, and cold stress (Chen et al. 2021; Gigli-Bisceglia et al. 2022). These latter 4 genes are located on the same region on the long arm of tomato chromosome 5. Although these results suggest a possible link between this chromosomal region and cold tolerance, there are other genes in the same genomic area which were not differentially regulated between the cold-tolerant and cold-sensitive RILs. Genes differentially expressed between the cold-tolerant and cold-sensitive RILs, before CS was applied, might be involved in pre-adaptation of plants to abiotic stress.

In fruits subjected to 2 h of CS, 10 genes were significantly differentially regulated between the cold-tolerant and cold-sensitive RILs (Fig. 1B). Of these, 4 genes exhibited higher expression in the cold-tolerant RILs, 3 of which were genes whose expressions were already higher in the cold-tolerant RILs before CS treatment, including Solyc05g013310 encoding for a receptor kinase, Solyc05g013150, encoding for lysine methyltransferase (LSMT), and Solyc05g013330, encoding for an RNA-binding protein; the 4th gene, Solyc09g020190, encoding for a non-specific phospholipase (SlNPC1), was upregulated only upon exposure to CS. It is worth noting, non-specific phospholipases (NPC) are recognized as key components of the phospholipid-signaling network involved in plant development and biotic and abiotic stress responses (Ali et al. 2022; Liu et al. 2023; Nakamura 2017). The remaining 6 DEGs exhibited higher expression in the cold-sensitive RILs upon exposure to CS. These included 1) Solyc05g 018050, showing homology to RING-type E3 ubiquitin ligases, which has been connected to improved plant survival under abiotic stresses (Al-Saharin et al. 2022). 2) Solyc05g005460, encoding for Nucleoredoxin 2 (SlNRX2), was previously shown to negatively regulate plant immunity (Cha et al. 2023); in tomato, SlNRX1 (Solyc05g005470) was shown to positively regulate heat stress tolerance by enhancing the transcription of antioxidants and heat-shock genes (Cha et al. 2022); NRXs are redox proteins that contain 3 tandemly arranged thioredoxin (TRX)-like modules and localized in both nucleus and cytoplasm (Kang et al. 2020); these proteins are potential nuclear TRXs found in most eukaryotic organisms (Marchal et al. 2014) and have been suggested to be master redox regulators of cell physiology and a hub of different redox-sensitive signaling pathways (Idelfonso-Garcia et al. 2022). 3) Solyc05g013450, exhibiting homology to multidrug and toxic compound extrusion (MATE) transporter detoxification-like proteins; MATE transporters perform various functions ranging from secondary metabolite transport to detoxification, disease resistance, and aluminum tolerance (Upadhyay et al. 2019). In Arabidopsis, MATE transporters DTX33 and DTX35 function as chloride channels essential for turgor regulation (Upadhyay, et al. 2019). Previously, expression of MATE detoxification-like genes was shown to be regulated by abiotic stresses in plants (Ali et al. 2021; Lu et al. 2018). 4) Solyc03g044790, encoding for methylesterase, methyl jasmonate-cleaving esterase; this enzyme was previously suggested to be a regulator of jasmonate signaling in plant (Stuhlfelder et al. 2004); for example, it’s been shown that the grapevine methylesterase 1 is significantly upregulated by cold or UV-B treatment, and is suggested to have a role in response to these stressors (Zhao et al. 2016). 5) Solyc09g059030, encoding for chloroplast envelope quinone oxidoreductase homolog (ceQORH); in Arabidopsis, for example, the plastidial protein ceQORH is an NADPH-dependent reductase whose activity may reduce long-chain, stress-related oxidized lipids. 6) Solyc09g018670, encoding for a protein with a TLC (TRAM, LAG1, and CLN8) lipid-sensing domain according to Prosite data site (https://prosite.expasy.org); the TLC domain is found in a family of membrane-associated proteins predicted to contain five transmembrane α helices (Si et al. 2019; Winter and Ponting 2002); although the role of the TLC domain is unknown, possible functions include involvement in lipid metabolism, sensing, or transport. In the present study, the LSMT encoding gene (Solyc05g013150) was upregulated in the cold-tolerant RILs, both before and 2 h following cold stress treatment. I a previous study, LSMT was identified to be highly induced 2 h following exposure of tomato fruits to 5 °C cold stress, but only in heat-treated fruit where the treatment reduced chilling injury development (Cruz-Mendívil et al. 2015a); this finding further supports a possible involvement of LSMT in postharvest fruit chilling tolerance.

It is possible that the genes highly expressed in the cold-tolerant RILs are involved in stress response and tolerance. Constitutively higher expression of such genes (without exposure to stress) may improve the coping of plants with cold stress. Genes that are highly expressed in the cold-sensitive RILs in response to CS, may activate biological processes required to nullify the negative effects of CS, such as elevated levels of toxic metabolites or ROS. The significant activation of gene expression observed in the cold-sensitive group after 24 h of CS exposure (Fig. 1C) may reflect the much lower ability of the cold-sensitive fruit to tolerate the stress, resulting in increased physiological and structural damages, which activate various protection pathways and relevant genes. Such responses (i.e. DEGs) could be mediated by calcium signaling and calcium ion binding proteins, which were upregulated more in the cold-sensitive fruit following 24 h exposure to CS (Fig. 1E). Previously, it was reported that calcium signaling activates and regulates several stress responses, including responses to CS (Iqbal et al. 2022). Calcium transport and signaling mechanisms are activated upon perception of CS, which induce responses to the stress in plant cells. These responses are mediated by specific sensors and activation of several transcription factors, leading to downstream gene expression and an appropriate response by the plant. In this context, calmodulin-binding proteins, which are highly represented among the DEGs following 24 h exposure to CS, are essential in regulating plant CS response.

The large set of genes (2454, Fig. 2) identified to be differentially regulated in all 6 RILs (3 cold tolerant and 3 cold sensitive) represents the core cold-response genes in tomato fruit, which probably are involved in stress adaptation and protection mechanisms. While specific transcriptome differences exist between the cold-tolerant and cold-sensitive RIL groups, considerable similarities in cold-response transcriptomes are also expected across the RILs due to their significant genomic similarities (average 50% identical by descent). The observation that across the 6 RILs photosynthesis-related genes in the fruit were negatively affected by the CS, suggests that syntheses and/or stabilities of the proteins responsible for binding chlorophyll molecules in the chloroplasts were reduced. These findings corroborate with prior reports that CS negatively impacted photosynthesis by affecting PSII activities (Allen and Ort 2001; Peng et al. 2015; Zhuang et al. 2019), which are particularly sensitive to CS. Similarly, our observation that circadian rhythm-related genes' expressions were altered by CS is in agreement with the previously-reported involvement of these genes in cellular protection, energy metabolism, and signaling pathways (Bieniawska et al. 2008; Sharma and Bhatt 2015). Our findings support possible involvement of circadian clock reprogramming in stress-related processes. In summary, the observed changes in gene expression in our investigation when fruit were exposed to CS are typical fruit responses to CS previously reported in other studies.

Differences were observed in starch and sugar metabolisms in the leaves of the cold-tolerant and cold-sensitive RILs (Fig. 5). The lower levels of starch and sucrose in the cold-tolerant RILs are likely related to the elevated glucose and fructose levels in these lines. The genetic make-up of the cold-tolerant RILs may significantly impact their starch metabolism, as evidenced by observed variation in starch accumulation in these RILs (Fig. 5). This observation implies that the cold-tolerant RILs may possess distinct genetic characteristic that impact their starch and sugar accumulation. Previously, sugars were suggested to be involved in sensitivity and response to postharvest chilling injury (Cao et al. 2013; Yu et al. 2022a), and surges in hexoses and soluble sugars in response to CS were suggested to contribute to cold tolerance (Dong and Beckles 2019, Tarkowski and Van den Ende 2015). Sugars are considered compatible solutes, which can protect sensitive membranes and proteins and increase cell turgor pressure to maintain cell volume. They also can function as ROS scavengers. Our findings further support the involvement of sugars in CS tolerance.

Most previous studies on plant response to CS included vegetative stage, though a few studies investigated CS responses during reproductive stage (Ding and Yang 2022). However, very few studies included both vegetative and reproductive stages, and the similarities or differences between molecular mechanisms contributing to CS during the two stages were not investigated. A few previous studies which investigated tomato fruit response to CS, identified some processes that were also common to vegetative stage response to CS; however, these studies also identified processes which were specific only to the fruit response to CS, including ripening-related and cell wall degradation-related gene expressions (Cruz-Mendívil et al. 2015a, 2015b; Hunter et al. 2021; Mitalo et al. 2023; Rugkong et al. 2011). Our study here indicates the presence of a positive correlation between the fruit chilling tolerance and cold tolerance in vegetative tissue in the investigated RILs. Specifically, the RILs that were identified with chilling tolerance in the fruit also exhibited cold tolerance in different vegetative stages, and RILs exhibiting chilling sensitivity in the fruit also exhibited cold-sensitivity in the vegetative tissue (Figs. 3 and 4). Molecular analyses further supported the presence of positive correlations in CS response between fruit and vegetative tissues; specifically, similar patterns of differential gene expressions were observed in fruit and leaves of cold-tolerant or cold-sensitive RILs (Fig. 6). These observations support the involvement of similar physiological and molecular responses to CS in the two stages, and suggest similarities in the way the two tissue types cope with the CS.

Postharvest storage of fruits and vegetables under low temperatures can prolong storing of crop produce, minimize crop loss, and improve farmers income. Chilling sensitivity of crops during postharvest storage, however, results in physiological injuries and subsequent pathogen infections, leading to major crop losses worldwide. While current approaches to mitigating chilling injuries during postharvest storage are reaching their limits, there are opportunities for improving plants’ chilling tolerance through genetic means and developing new crop cultivars with better low temperature tolerance during postharvest storage (Albornoz et al. 2022). The present study provides evidence of the presence of CS tolerance in tomato fruit, and identified genes, physiological and biochemical processes associated with fruit chilling tolerance. These findings may be used as genetic/physiological/biochemical markers to breed new tomato inbred lines and hybrid cultivars with improved postharvest chilling tolerance. The identified genes may also provide the necessary information for developing transgenic tomato plants with chilling tolerance during postharvest storage. Further, the findings that similar genes might contribute to cold/chilling tolerance during seedling/early-vegetative stages and postharvest fruit indicate the possibility of rapid screening for cold tolerance during early vegetative stages and develop germplasm with improved chilling tolerance during postharvest fruit storage.

## Materials and methods

### Plant material

A tomato recombinant inbred line (RIL) population (*n* = 148 lines), previously developed from an interspecific cross between *Solanum pimpinellifolium* L. accession LA 2093 and *S. lycopersicum* L. breeding line NC EBR1 (Ashrafi, et al. 2009) and subsequently genetically mapped with more than 144,000 SNP markers (Gonda, et al. 2019), was screened in our earlier studies to determine the extent of genetic variation in fruit chilling tolerance during postharvest storage; several RI lines with extreme response to chilling stress were selected and physiologically characterized (David, et al. 2022). Pictures of the tomato fruits exhibiting the different responses to the chilling stress following postharvest cold storage were previously presented (David et al. 2022) and are displayed here in Fig S6 (adopted from David et al 2022). In the present study, we selected extreme groups, the highly cold/chilling tolerant (i.e., tolerant class) and highly cold/chilling sensitive (i.e., sensitive class), for further studies. The “cold tolerant” class included RILs 47, 49, 65 and 99, and the “cold sensitive” class included RILs 5, 71, 90, 135 and 150, as reported previously (David, et al. 2022). A schematic representation of the experimental setup is included in Supplementary Fig. S1.

### Plant growth and fruit tissue sampling for investigating fruit chilling stress transcriptomic response

Three RI lines from each of the tolerant class (RILs 47, 65 and 99) and sensitive class (RILs 71, 135 and 150) were grown in pots containing artificial soil medium (Green 77 artificial soil, Even-Ari Ltd., Beit Elazari, Israel) in a greenhouse (GH) at the Volcani Institute, Rishon LeZion, Israel, with day temperature of ~ 25–30 °C (and natural light) and night temperature of ~ 17–23 °C. Mature-green (MG) stage fruit were harvested in the morning from at least 3 plants of each RI line. Efforts were made to harvest fruit of similar size, shape and maturity, and fruit calyxes were removed after harvest. Immediately after harvest, fruit were exposed to low temperature of 1.5 °C for 2 or 24 h in an incubator. Subsequently, fruit pericarp tissue was collected from cold-stressed fruit, and freshly-harvested fruit (non-chilled control), immediately flash-frozen under liquid nitrogen (LN), and stored at -80 °C for later use in RNA–seq transcriptomic analysis (described below).

### Plant growth and vegetative tissue sampling for investigating vegetative stage chilling stress response

Several experiments were conducted to investigate the effects of CS (1.5 °C) on tomato plants during vegetative stage, as described below.

#### Experiment 1

To study general effects of CS on leaf tissue, plants of the “cold-tolerant” (RILs 47, 65 and 99) and “cold-sensitive” class (RILs 71, 135 and 150) were grown in a temperature-controlled GH at 25 °C, with 16-h-light/8-h-dark natural light. These plants were grown in 500-ml pots filled with perlite, and regularly fertigated (Shaphir Or M4-2–6 + 6%, Gat Fertilizers Ltd, Kiryat Gat, Israel). One-month-old plants were exposed to 1.5 °C for 3 d, and leaf tissues were sampled for measuring electrolyte leakage and Malondialdehyde (MDA) content (described below).

#### Experiment 2

To study physiological, biochemical and molecular responses of plants to CS, plants of the “cold-tolerant” (RILs 47, 49, 65 and 99) and “cold-sensitive” class (RILs 5, 71, 90 and 150) were grown in pots containing natural soil in a temperature-controlled GH at 25 °C, 16-h-light/8-h-dark natural light. One-month-old plants were exposed to low temperature of 1.5 °C for 24 h, after which they were returned to the GH set at 25 °C and kept for 10 days. Leaf tissues for gene expression analyses were collected after 2 and 24 h exposure to CS; leaf tissues for assessing leaf injury and electrolyte leakage were collected after 2 d exposure to CS, and leaf tissues for H_2_O_2_ and MDA measurements were collected after 24 h exposure to CS (described below). Further, plant survival was visually assessed after 10-d recovery at 25 °C.

#### Experiment 3

Effects of CS on 10-d-young seedlings were also investigated. For this experiment, seed of the “cold-tolerant” (RILs 47, 49, 65 and 99) and “cold-sensitive” class (RILs 5, 71, 90 and 150) were plated onto Petri dishes containing ½-strength MS medium and maintained in dark at room temperature; following seed germination, plates were transferred to a growth incubator set at 25 °C and 16-h-light/8-h-dark, and subsequently, the 10-d-young seedlings were exposed to CS of 1.5 °C for 24 h, followed by recovery for 7 d at 25 ºC (16-h-light/8-h-dark). Tissues were sampled for electrolyte leakage and MDA content measurements immediately after 24 h CS exposure (described below); seedling survival was visually assessed after 7-d recovery at 25 °C during which chilling injury consequences to leaf and plant survival is manifested.

### Vegetative tissue collection for RNA extraction and various biochemical analyses

For RNA extraction, approx. 100 mg of the 3rd leaf was collected from one-month-old plants that were exposed to CS (Exp. 1 above); samples were pooled from 4 plants per RI line to yield one replicate, with a total of 3 biological replicates per RIL. For the biochemical analyses, approx.1 g of the 3rd leaf was collected from 4 plants of each RI line and pooled to yield one biological replicate, with a total of 4 biological replicates per RIL (Exp. 2 above). For the MS-grown 10-d-young seedlings (Exp. 3 above), each biological replicate per RIL included a pool of 6 whole seedlings; this process was repeated 4 times to obtain 4 biological replicates per RIL. For all vegetative tissue analyses, the collected tissue samples were flash-frozen in LN and stored at -80 ºC. Subsequently, the samples were lyophilized for 5 days and ground under LN into a fine powder using a Geno/Grinder®—Automated Tissue Homogenizer and Cell Lyser; powdered material were stored at -80 ºC until analysis.

### Chilling injury assay and measurement of total chlorophyll content

The severity of chilling injury (CI) was assessed visually, mainly based on the level of leaf tissue dehydration and development of tissue surface injury on a scale of 0–2, where 0 = no visual damage, 1 = medium damage, covering < 50% of the leaf surface, and 2 = severe damage, (covering 50–100% of the leaf surface). The CI index was calculated as:

$$CI\;index=\Sigma\;(CI\;level\;\ast\;number\;of\;leaves\;at\;the\;CI\;level)\;/\;total\;number\;of\;leaves\;in\;the\;treatment$$

Survival of soil-grown plants (*Exp. 2*) or MS-medium-grown seedlings (*Exp. 3*) exposed to CS (1.5 °C) was measured after being transferred to normal (25 ºC) conditions with 16/8 h day/night for 10 or 7 days, respectively. To measure total chlorophyll content, 10 mg of the lyophilized samples were dissolved in 10 mL of 80% ETOH and stored for 48 h, and chlorophyll content was measured, as described elsewhere (Camalle et al. 2020).

### Electrolyte leakage assay

Two days after exposure to CS, 2 leaflets from the 3rd leaf of the soil-grown plants (Exp. 2), or 2 seedlings from the MS-medium-grown seedlings (Exp. 3), were soaked in 40 mL of dH_2_O and stored overnight at room temperature, and the initial electroconductive value (*Ci*) resulting from ion leakage was measured. Subsequently, samples were autoclaved for 30 min, cooled to room temperature for 4 h, and the total electroconductive value (*Cm*) was measured. The electrolyte leakage index was calculated as: *Ci*/*Cm* × *100*.

### Hydrogen peroxide content determination

Hydrogen peroxide (H_2_O_2_) content was extracted from 5 mg of lyophilized leaf tissue of the soil-grown plants (Exp. 2), as described elsewhere (Yesbergenova et al. 2005) with some modifications. Briefly, 600 µL of extraction buffer (containing 200 mM HCl) was added to the powdered tissue, the homogenate was centrifuged at 12,000 RPM for 15 min at 4ºC, an aliquot of 500 µL supernatant was neutralized by adding 100 µL of 50 mM phosphate buffer (P-buffer) pH 7.5 and 400 µL of 200 mM NaOH, and the mixture was vortexed for 10 s. The reaction mixture used comprised 90 µL of 50 mM Tris–HCL buffer pH 6.5, 20 µL of 8.5 mM 4-aminoantipyrine, 20 µL of 3.4 mM sodium 3, 5- dichloro-2-hydroxybenzenesulfonate, 50 µL of neutralized supernatant, and 20 µL of 45 U/ml horseradish peroxidase. H_2_O_2_ content was measured by reading absorbance at 515 nm, using a BioTek Synergy H1 microplate reader, (BioSPX; Abcoude, The Netherlands). Calculation of H_2_O_2_ was based on calibration against a standard curve with H_2_O_2_ known concentrations from 0–200 nM.

### Malondialdehyde content determination

Malondialdehyde (MDA) content, indicative of chilling-induced lipid peroxidation, was quantified in the soil-grown plants (Exp. 2, 3rd leaf) and the MS-medium-grown seedlings (Exp. 3, shoots), as described elsewhere (Van Hasselt 1974) with minor modifications. Briefly, 5 mg of the lyophilized tissue was extracted with 1 mL of chilled phosphate-buffered saline containing 10% (w/v) trichloroacetic acid and 0.01 mM phenylmethylsulfonyl fluoride. Following centrifugation at 13,000 RPM for 15 min at 4 ºC, the supernatant was collected, mixed with one volume of 5% 2-thiobarbituric acid, and incubated for 45 min at 80 ºC. Malondialdehyde content was determined spectrophotometrically using 200 µL of the supernatant at 440, 532 and 600 nm wavelengths, and calculated as nmol g⁻¹ DW.

### Starch analysis

Lugol's iodine staining reagent (Sigma; Rehovot, Israel) was used to visualize starch. Leaves were collected from one-month-old soil-grown plants (Exp. 2) before and after CS. Leaves were boiled at 90 ºC with 80% ETOH for 20 min, rinsed with ddH_2_O, stained with Lugol's reagent, and briefly washed with water.

### Sugar content determination

Total Sugars were extracted from 20 mg of lyophilized tissue using 80% ETOH at 80 ºC in a water bath for 45 min (repeated 3 times). The ETOH from the pooled supernatants was evaporated in a CentriVap Concentrator (Lancoco Kansas City, MO, USA), and dried samples were re-suspended in 1 mL Mili-Q water and filtered through a 0.2 µm membrane. The filtered solution was used for quantification of sucrose, glucose and fructose by ultra-fast liquid chromatography (UFLC), as described elsewhere (Teper-Bamnolker et al. 2023).

### RNA extraction and sequencing (RNA-seq)

Pericarp tissues from 5 fruit, or leaf tissues from 4 plants, per each selected RIL were pooled to yield one biological replicate. Samples were ground in LN using a mortar and pestle. Three biological replicates per RIL were used for each of the fruit and leaf analyses. Total RNA was extracted using spectrum™ total RNA kit (Sigma Aldrich, St. Louis, MO, USA) according to the manufacturer's instructions. The quality and quantity of the extracted RNAs were assessed by agarose gel electrophoresis, as well as a Thermo NanoDrop 2000 spectrometer (Thermo Fisher Scientific, Wilmington, NC, USA). For performing fruit transcriptomic analyses, library preparation and RNA-sequencing were carried out at the NGS Macrogen Europe company (Amsterdam, The Netherlands). Total RNA concentration was determined using a Quant-IT RiboGreen (Invitrogen, Carlsbad, CA, USA). Samples were run on a TapeStation RNA screentape (Agilent, Santa Clara, CA, USA) to assess the integrity of the RNA. Only high-quality RNA extracts with RIN greater than 7.0 were used for RNA library construction.

A library was constructed composed of one µg of total RNA from each sample by Illumina TruSeq mRNA Sample Prep kit (Illumina, Inc., San Diego, CA, USA). The first step in the workflow involved purifying the poly-A-containing mRNA molecules using poly‐T oligo‐attached magnetic beads. Following purification, the mRNA was fragmented into small pieces using divalent cations under elevated temperatures. The cleaved RNA fragments were reverse-transcribed into first-strand cDNA using SuperScript II reverse transcriptase (Invitrogen, Carlsbad, CA, USA) and random primers. Second-strand cDNA synthesis was performed using DNA Polymerase I and RNase H. Subsequently, the cDNA fragments were taken through an end repair process, adding a single 'A' base, and ligated to indexing adapters. The products were then purified and enriched with PCR to create the final cDNA library. The libraries were quantified using qPCR according to the qPCR Quantification Protocol Guide (KAPA Library Quantification kits for Illumina Sequencing platforms, San Diego, CA, USA) and quantified using a TapeStation D1000 ScreenTape (Agilent Technologies, Waldbronn, Germany). Indexed libraries were then submitted to Illumina NovaSeq (Illumina, Inc., San Diego, CA, USA), where the paired-end (2 × 100 bp) sequencing was performed.

For RT-qPCR analysis, cDNA was synthesized from purified RNA using the high-capacity cDNA reverse transcription kit (Applied Biosystems, Waltham, MA, USA). The generated cDNA was diluted 10 or 100 times, and quantitative analysis of transcripts performed by employing a set of specific primers (Supplementary Table 1). The tomato clathrin AP-2 complex subunit (*CAC*) was used as a reference gene (Gonzalez-Aguilera et al. 2016). The RT-qPCR analysis was performed with a 10 µL reaction mixture prepared with 5 µL of Power SYBR® Green PCR Master Mix (Applied Biosystems 7500, Waltham, MA, USA), 0.3 µL of primers, 2 µL of cDNA and 2.7 µL of DNase-free water. Amplifications were monitored in RT-qPCR using Applied Biosystems version 2.2.2. Three biological replicates for each sample were normalized to the *CAC* reference gene (ΔCt = Ctgene tested – Ct*CAC*). All data were expressed as an n-fold change of gene expression.

### Bioinformatics and statistical analyses

A total of ~ 1.13 billion paired-end reads (with an average of 21 million per each sequenced sample) were mapped to the reference genome of *S. lycopersicum* (ftp://ftp.solgenomics.net/tomato_genome/assembly/build_4.00/) using Tophat2 software, as described elsewhere (Kim et al. 2013). Gene abundance was estimated using Cufflinks v. 2.2 software program (Trapnell et al. 2010) combined with gene annotations from the Sol Genomics Network (SGN) database (https://solgenomics.net/; ftp://ftp.solgenomics.net/tomato_genome/annotation/ITAG4.0_release). Gene expression values were computed as FPKM (Fragments Per Kilobase of transcript per Million mapped reads). Principal component analysis (PCA) was carried out using R Bioconductor (Gentleman et al. 2004). Differential gene expression analysis was performed with the DESeq2 R package (Love et al. 2014). Genes that were ≥ twofold differentially expressed (DE) with a false discovery-corrected statistical significance of *p* < 0.05 were considered differentially expressed (Benjamini and Hochberg 1995). Cluster analysis of the significant DE genes in each RIL, based on the average FPKM value, was conducted using Expander 7 software (Ulitsky et al. 2010) with the K-means algorithm (Shamir et al. 2005). Gene Ontology (GO) enrichment analysis (at *p* < 0.05) of the differentially expressed genes (DEGs) was performed using Panther (http://www.pantherdb.org/) and KOBAS tool (http://bioinfo.org/kobas/genelist). The figures of enrichment results displayed in bubble gradient, and heatmaps were generated by SRplot (https://www.bioinformatics.com.cn/srplot).

Four biological replicates were analyzed, consisting of either 4 of the soil-grown plants (Exp. 2) or 6 of the MS-grown seedlings (Exp. 3). Data were analyzed by *t*-test, or one-way ANOVA, followed by multiple comparison tests with *Tukey's* HSD post-hoc test at *p* < *0.05*, using the JPM Pro 16 statistical package and *R* Program. The bar plots were generated using *R* software by the ggplot2 package (https://ggplot2.tidyverse.org).

## Supplementary Information

Supplementary Material 1: Supplementary Figure S1. Schematic representation of experimental set up. Transcriptomic and bioinformatic analyses were performed for fruits of RILs before (0 h), 2 h, and 24 h following exposure to postharvest 1.5 °C cold stress. Physiological, biochemical, and molecular analyses were performed for soil and MS-grown plants before (0 h), 2 and 24 h following exposure to cold 1.5 °C stress. Supplementary Figure S2. PCA analysis of the transcriptomic data. Data points of gene expression from the transcriptomic analysis were subject to PCA analysis. Data for 24 h following exposure to cold stress is labeled: Each of the three-cold tolerant RILs (47, 65, 99) are encircled with a red oval, and data points for all three-cold tolerant RILs are encircled with a dotted red oval. Similar labeling for cold-sensitive RILs (71, 135, 150) is shown with blue ovals. Supplementary Figure S3. Exposure of different RIL plants to cold stress following recovery growth. Pictures of soil-grown plants following exposure to cold stress of 1.5 °C for two days followed by 10 days of growth recovery at 25 °C. (A) Cold-sensitive (5, 71, 90, 150) and (B) cold-tolerant (47, 49, 65, 99) RILs. Supplementary Figure. S4. The impact of cold treatment on chilling injury parameters, electrolyte leakage and MDA parameters. (A) Electrolyte leakage and (B) MDA values were measured in leaves of cold-sensitive (71, 135, 150) and cold-tolerant (47, 65, 99) perlite-grown plants following exposure to cold stress of 1.5 °C for 3 days. Data are means ± SE, *n*=4; biological replicates. Different lowercase letters indicate significant differences between sensitive and tolerant lines. One-way ANOVA *p≤* 0.05, as determined by Turkey-Kramer HSD. Supplementary Figure S5. The starch levels in RILs categorized as (A) cold-sensitive (71, 135, and 150) and (B) cold-tolerant (47, 65, and 99) before cold stress. Supplementary Figure S6. *Adopted from David et al,* 2022*.* Development of surface chilling injuries in RIL fruit following postharvest cold storage. Examples for fruits from different tomato RILs, four tolerant RILs (left panel), and four sensitive RILs (right panel). Fruits at the MG ripening stage were harvested and immediately stored for 14 days at 1.5°C followed by three days at 20 °C.

Supplementary Material 2: Supplementary Table S1. Tomato primer sequences used for real-time quantitative reverse transcriptase polymerase chain reaction (qRT-PCR). Supplementary Table S2. DEG between cold tolerant and cold sensitive RILs before application of cold stress. Supplementary Table S3. DEG between cold tolerant and cold sensitive RILs 2 h following application of cold stress. Supplementary Table S4. Data set comparing gene expression in fruits of the six studied RILs at 0, 2, and 24 h following postharvest cold stress. Supplementary Table S5. Genes associated with calcium mediated signaling differentially expressed between cold tolerant and cold sensitive RILs following 24 h of cold stress. Supplementary Table S6. Data set including differential gene expression in fruits following exposure to cold stress common to all six RIL genotypes. Supplementary Table S7. Genes associated with *Response to Heat Stress* GO term downregulated in all 6 RILs after 24 h of cold stress. Supplementary Table S8. Regulatory genes included in cluster 2 Biological Process GO term *Responses to Stimuli* gradually induced in all RILs following cold stress. Supplementary Table S9. Heat shock protein (Hsps) genes included in cluster 5 Biological Process GO term *Protein Folding* transiently induced after 2 h of cold stress.

## Data Availability

The datasets generated and analyzed during the current study are available in the Sequence Read Archive (SRA), Biological Research Project Data (BioProject), National Center for Biotechnology Information (NCBI) repository, accession: PRJNA1078083.

## Abbreviations

CI: Chilling injury
CS: Cold stress
GH: Greenhouse
LN: Liquid nitrogen
MDA: Malondialdehyde
MG: Mature-green
PCA: Principal component analysis
RIL: Recombinant inbred line
