# Supplementary material for: Molecular and biochemical components associated with chilling tolerance in tomato: comparison of different developmental stages
Source: Mol Hortic. 2024 Sep 5;4:31. doi: 10.1186/s43897-024-00108-0 (PMC11375913; doi:10.1186/s43897-024-00108-0)
Supplement: Supplementary file 1 — Supplementary Material 1: Supplementary Figure S1. Schematic representation of experimental set up. Transcriptomic and bioinformatic analyses were performed for fruits of RILs before (0 h), 2 h, and 24 h following exposure to postharvest 1.5 °C cold stress. Physiological, biochemical, and molecular analyses were performed for soil and MS-grown plants before (0 h), 2 and 24 h following exposure to cold 1.5 °C stress. Supplementary Figure S2. PCA analysis of the transcriptomic data. Data points of gene expression from the transcriptomic analysis were subject to PCA analysis. Data for 24 h following exposure to cold stress is labeled: Each of the three-cold tolerant RILs (47, 65, 99) are encircled with a red oval, and data points for all three-cold tolerant RILs are encircled with a dotted red oval. Similar labeling for cold-sensitive RILs (71, 135, 150) is shown with blue ovals. Supplementary Figure S3. Exposure of different RIL plants to cold stress following recovery growth. Pictures of soil-grown plants following exposure to cold stress of 1.5 °C for two days followed by 10 days of growth recovery at 25 °C. (A) Cold-sensitive (5, 71, 90, 150) and (B) cold-tolerant (47, 49, 65, 99) RILs. Supplementary Figure. S4. The impact of cold treatment on chilling injury parameters, electrolyte leakage and MDA parameters. (A) Electrolyte leakage and (B) MDA values were measured in leaves of cold-sensitive (71, 135, 150) and cold-tolerant (47, 65, 99) perlite-grown plants following exposure to cold stress of 1.5 °C for 3 days. Data are means ± SE, n=4; biological replicates. Different lowercase letters indicate significant differences between sensitive and tolerant lines. One-way ANOVA p≤ 0.05, as determined by Turkey-Kramer HSD. Supplementary Figure S5. The starch levels in RILs categorized as (A) cold-sensitive (71, 135, and 150) and (B) cold-tolerant (47, 65, and 99) before cold stress. Supplementary Figure S6. Adopted from David et al, 2022. Development of surface chilling [file 43897_2024_108_MOESM1_ESM.pptx]

## Slide 1
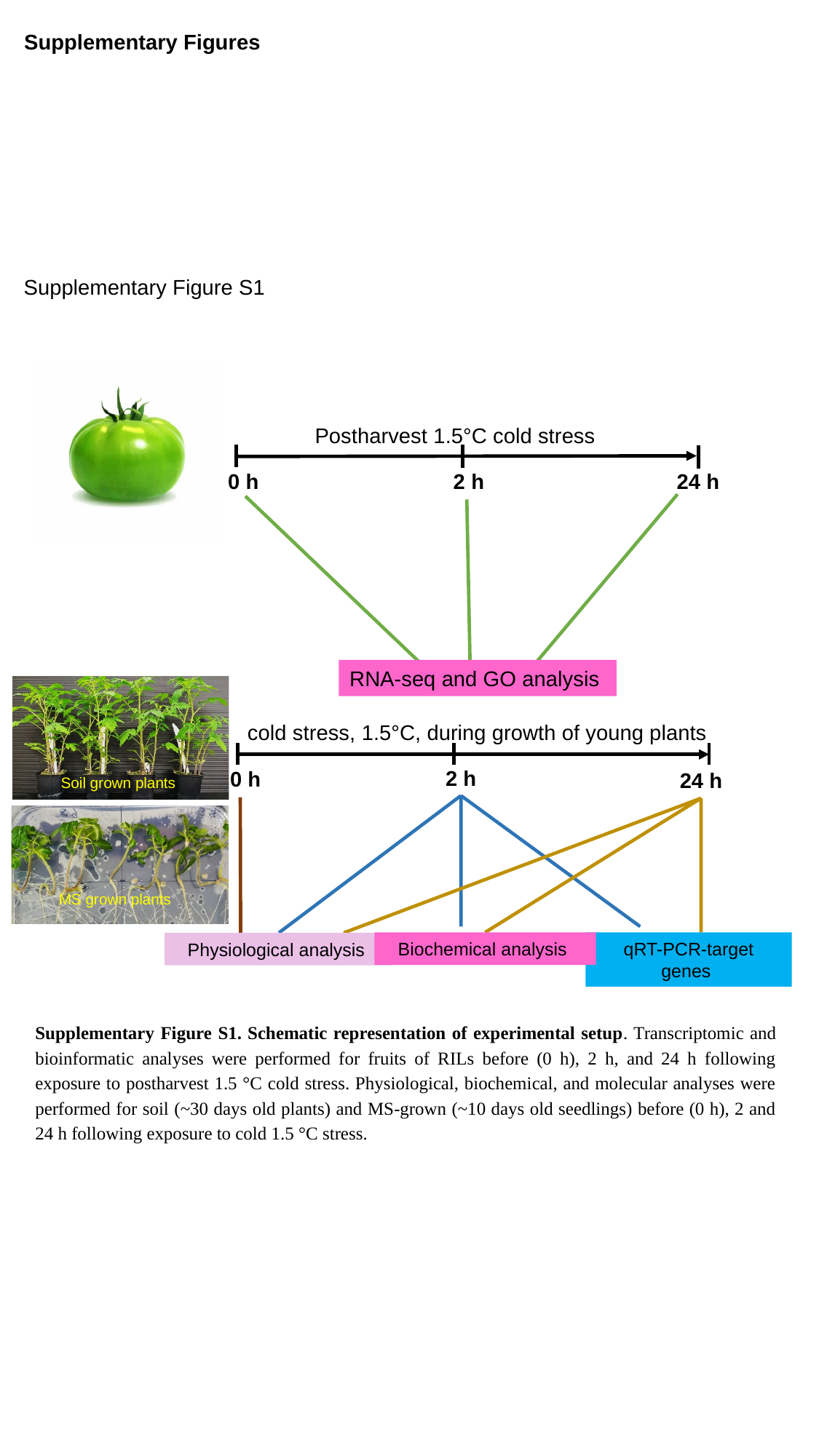

Supplementary Figures
Supplementary Figure S1
0 h
2 h
24 h
RNA-seq and GO analysis
Postharvest 1.5°C cold stress
cold stress, 1.5°C, during growth of young plants
2 h
0 h
24 h
Soil grown plants
MS grown plants
Biochemical analysis
qRT-PCR-target genes
Physiological analysis
Supplementary Figure S1. Schematic representation of experimental setup. Transcriptomic and bioinformatic analyses were performed for fruits of RILs before (0 h), 2 h, and 24 h following exposure to postharvest 1.5 °C cold stress. Physiological, biochemical, and molecular analyses were performed for soil (~30 days old plants) and MS-grown (~10 days old seedlings) before (0 h), 2 and 24 h following exposure to cold 1.5 °C stress.

## Slide 2
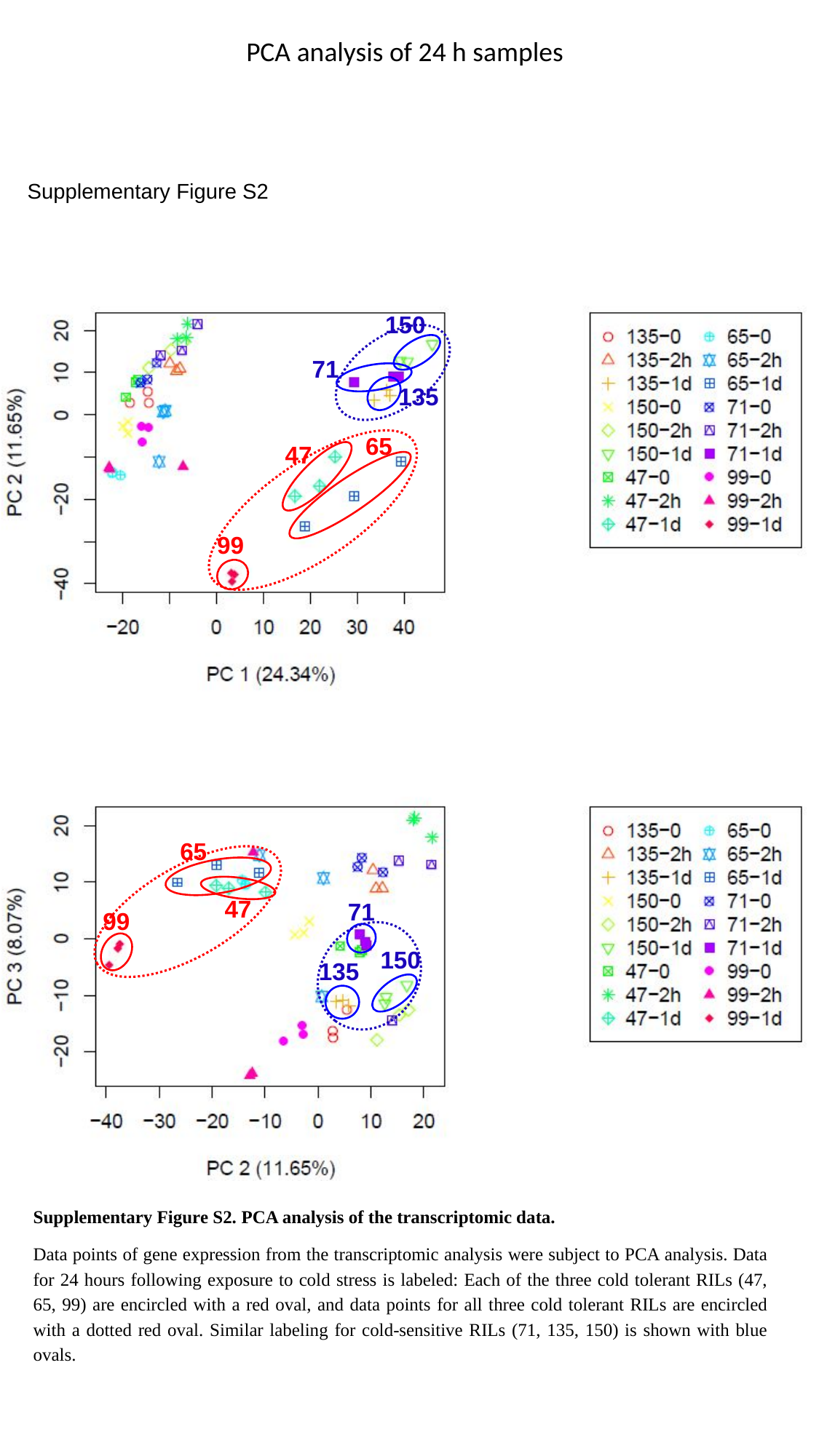

PCA analysis of 24 h samples
Supplementary Figure S2
150
71
135
65
47
99
65
47
71
99
150
135
Supplementary Figure S2. PCA analysis of the transcriptomic data.
Data points of gene expression from the transcriptomic analysis were subject to PCA analysis. Data for 24 hours following exposure to cold stress is labeled: Each of the three cold tolerant RILs (47, 65, 99) are encircled with a red oval, and data points for all three cold tolerant RILs are encircled with a dotted red oval. Similar labeling for cold-sensitive RILs (71, 135, 150) is shown with blue ovals.

## Slide 3
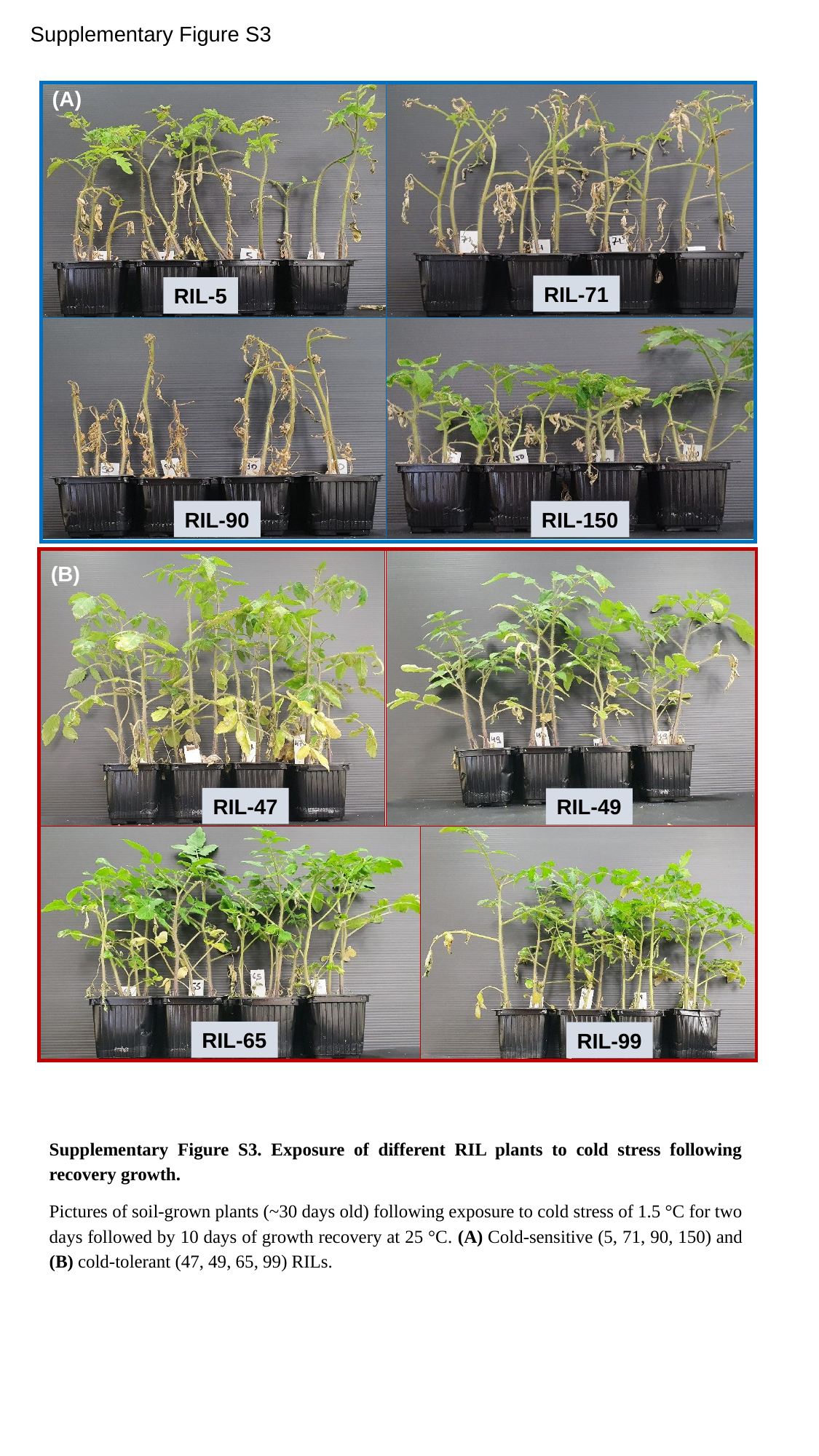

Supplementary Figure S3
(A)
RIL-71
RIL-5
RIL-90
RIL-150
(B)
RIL-47
RIL-49
RIL-65
RIL-99
(C)
Supplementary Figure S3. Exposure of different RIL plants to cold stress following recovery growth.
Pictures of soil-grown plants (~30 days old) following exposure to cold stress of 1.5 °C for two days followed by 10 days of growth recovery at 25 °C. (A) Cold-sensitive (5, 71, 90, 150) and (B) cold-tolerant (47, 49, 65, 99) RILs.

## Slide 4
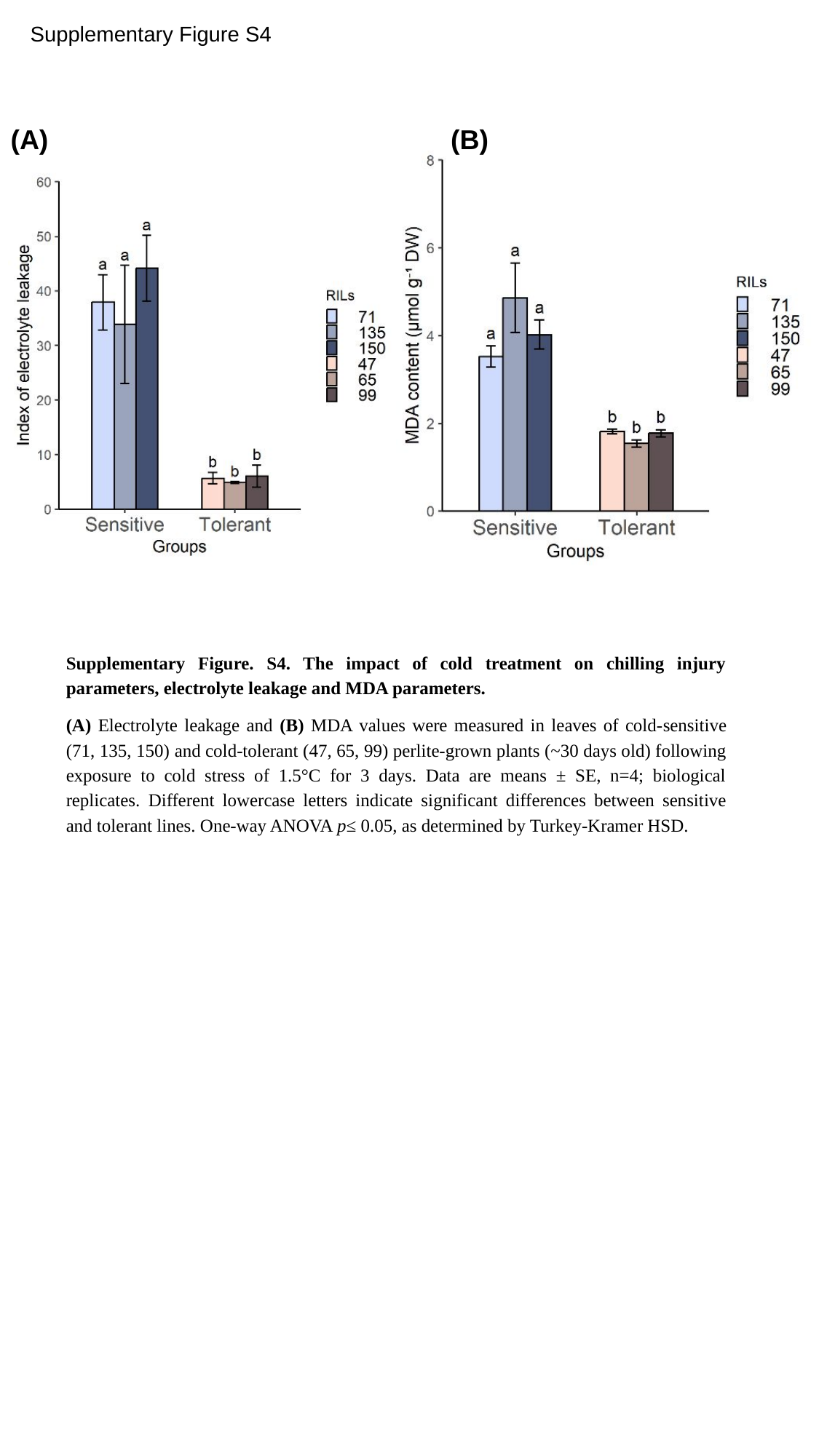

Supplementary Figure S4
(A)
(B)
Supplementary Figure. S4. The impact of cold treatment on chilling injury parameters, electrolyte leakage and MDA parameters.
(A) Electrolyte leakage and (B) MDA values were measured in leaves of cold-sensitive (71, 135, 150) and cold-tolerant (47, 65, 99) perlite-grown plants (~30 days old) following exposure to cold stress of 1.5°C for 3 days. Data are means ± SE, n=4; biological replicates. Different lowercase letters indicate significant differences between sensitive and tolerant lines. One-way ANOVA p≤ 0.05, as determined by Turkey-Kramer HSD.
(C)

## Slide 5
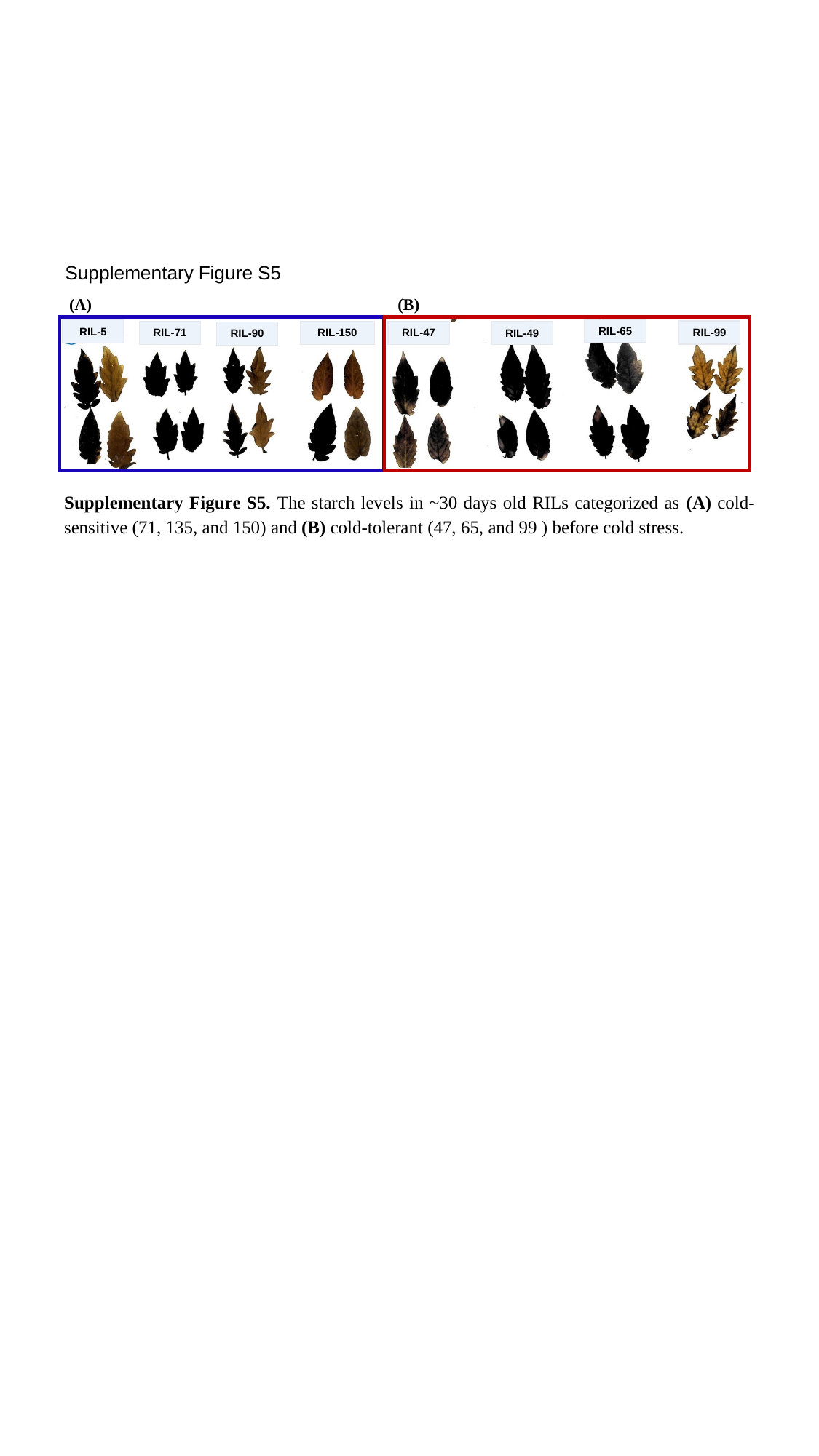

Supplementary Figure S5. The starch levels in ~30 days old RILs categorized as (A) cold-sensitive (71, 135, and 150) and (B) cold-tolerant (47, 65, and 99 ) before cold stress.

## Slide 6
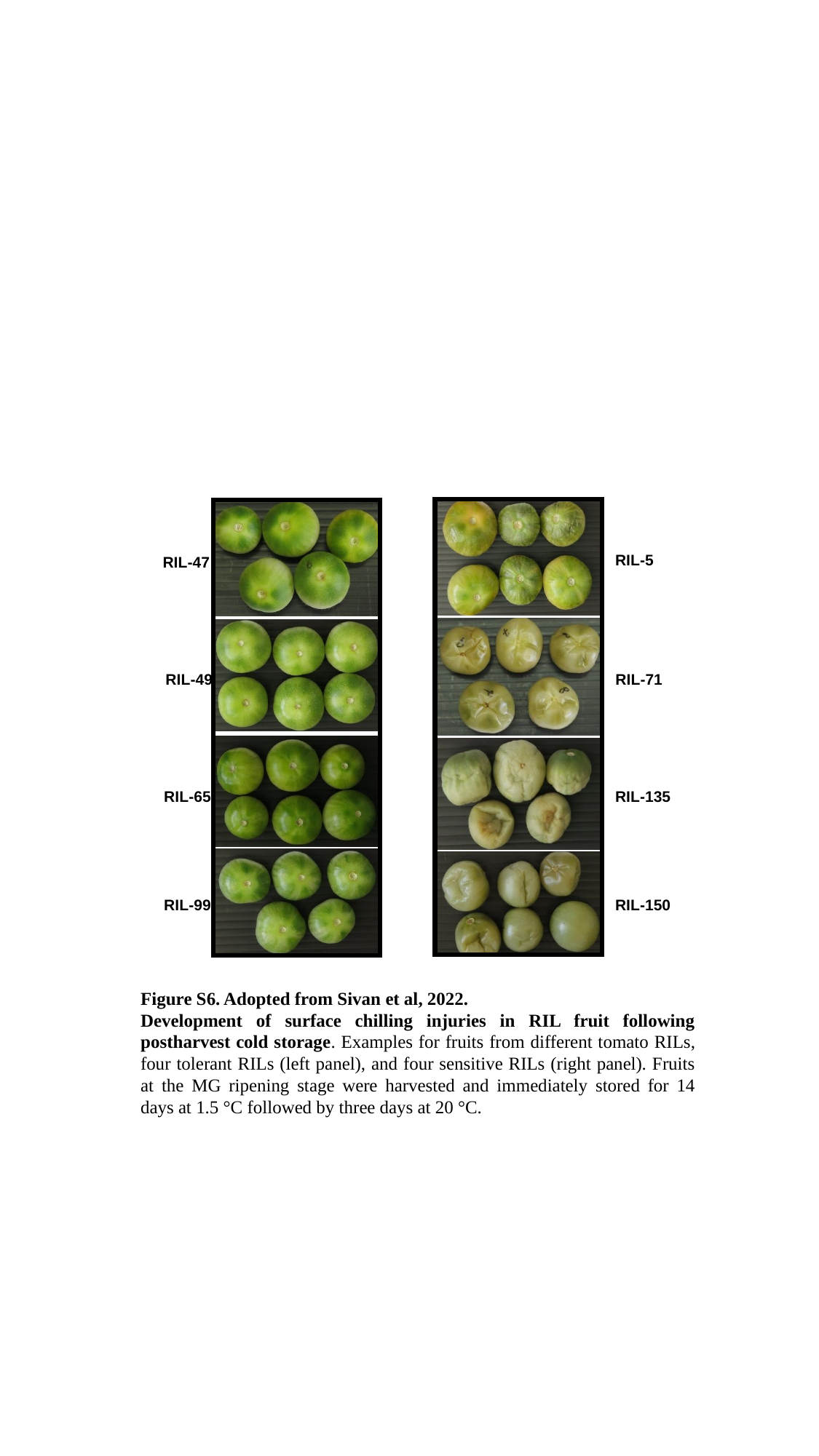

RIL-5
RIL-47
RIL-71
RIL-49
RIL-135
RIL-65
RIL-99
RIL-150
Figure S6. Adopted from Sivan et al, 2022.
Development of surface chilling injuries in RIL fruit following postharvest cold storage. Examples for fruits from different tomato RILs, four tolerant RILs (left panel), and four sensitive RILs (right panel). Fruits at the MG ripening stage were harvested and immediately stored for 14 days at 1.5 °C followed by three days at 20 °C.
