# Supplementary material for: Molecular and biochemical components associated with chilling tolerance in tomato: comparison of different developmental stages
Source: Mol Hortic. 2024 Sep 5;4:31. doi: 10.1186/s43897-024-00108-0 (PMC11375913; doi:10.1186/s43897-024-00108-0)
Supplement: Supplementary file 2 — Supplementary Material 2: Supplementary Table S1. Tomato primer sequences used for real-time quantitative reverse transcriptase polymerase chain reaction (qRT-PCR). Supplementary Table S2. DEG between cold tolerant and cold sensitive RILs before application of cold stress. Supplementary Table S3. DEG between cold tolerant and cold sensitive RILs 2 h following application of cold stress. Supplementary Table S4. Data set comparing gene expression in fruits of the six studied RILs at 0, 2, and 24 h following postharvest cold stress. Supplementary Table S5. Genes associated with calcium mediated signaling differentially expressed between cold tolerant and cold sensitive RILs following 24 h of cold stress. Supplementary Table S6. Data set including differential gene expression in fruits following exposure to cold stress common to all six RIL genotypes. Supplementary Table S7. Genes associated with Response to Heat Stress GO term downregulated in all 6 RILs after 24 h of cold stress. Supplementary Table S8. Regulatory genes included in cluster 2 Biological Process GO term Responses to Stimuli gradually induced in all RILs following cold stress. Supplementary Table S9. Heat shock protein (Hsps) genes included in cluster 5 Biological Process GO term Protein Folding transiently induced after 2 h of cold stress. [file 43897_2024_108_MOESM2_ESM.zip › Supplementary Table S1 7-4-2024.docx]

**Supplementary Table S1**: Tomato primer sequences used for real-time quantitative reverse transcriptase polymerase chain reaction (qRT-PCR)

|  |  |  |
| --- | --- | --- |
| Gene | ID | Primer sequence (5'-3') |
|  |  |  |
| Clathrin AP-2 complex subunit (*CAC*) | Solyc08g006960 | F: CCTCCGTTGTGATGTAACTGG |
|  |  | R: ATTGGTGGAAAGTAACATCATCG |
| Lysine methyltransferase | Solyc05g013150 | F: AGCAGAGCAAACGAAGAG |
|  |  | R: CCCTCCCTTCATCAGTTTC |
| Receptor kinase 1 | Solyc05g013310 | F: AATGGGCAAAGGAGTGTC |
|  |  | R: GCATTTCACCGCTGTTTC |
| Stress associated RNA-binding protein | Solyc05g013330 | F: ACATACAGAGAGAGGGATGG |
|  |  | R: AGGAGATGAGGCTTCTTACA |
| Receptor kinase 2 | Solyc05g013320 | F: TTTGGAGAAACAGCGGTAAA |
|  |  | R: CTTGGAGACGAAGTGCATAC |
| Calmodulin-like 37 | Solyc11g071750 | F: AGAGGAATAAAGAGAGTGAGTTG |
|  |  | R: TCTTCAAGCTCTTAGGAGTAATG |
| Calcium-transporting ATPase | Solyc02g092450 | F: TTACTGGTGACGGAACTAATG |
|  |  | R: TGATTGGGAATGGACTAACG |
| Calcium-dependent lipid-binding | Solyc01g099370 | F: CTCACGAAATCAGGGACTTAC |
|  |  | R: GGAACAGATACCTCACCAATATC |
| Ethylene Response factor D.4 | Solyc10g050970 | F: GGTTGCTGCTTTAACCAATG |
|  |  | R: TACTGACAACGCTCTGTTTC |
